# APOLIPOPROTEIN E, LEUKOCYTE TELOMERE LENGTH AND MEMORY IN EXCEPTIONALLY LONG-LIVED FAMILIES

**DOI:** 10.1093/geroni/igz038.3446

**Published:** 2019-11-08

**Authors:** Adiba Ashrafi, Megan Barker, Larry Honig, Joseph Lee, Stacy L Andersen, Joseph M Zmuda, Mary K Wojczynski, Stephanie Cosentino

**Affiliations:** 1 Columbia University, New York, New York, United States; 2 Boston University School of Medicine, Boston, Massachusetts, United States; 3 Department of Epidemiology, University of Pittsburgh; Pittsburgh, Pennsylvania, United States; 4 Department of Genetics, Washington University in St. Louis, Saint Louis, Missouri, United States

## Abstract

Exceptional aging has heritable components. One genetic risk factor for cognitive aging may be Apolipoprotein E (APOE), but it is unclear to what extent APOE relates to cognitive aging versus risk of Alzheimer’s disease. Cognitive aging may also be influenced by leukocyte telomere length (LTL), posited to be a marker of “biological age”. We examine the relationship between APOE, LTL, and memory in aging. For APOE, effects of ε4 (ε3ε4/ε4ε4) and ε2 (ε2ε3/ε2ε2) versus the more common ε3ε3 referent genotype on episodic (EM) and working memory (WM) were examined, comparing longevous families to the general population. Participants belonged to a multi-generational, international cohort (Long Life Family Study) including relatives from long-lived families and spouse-controls. 3,654 participants with valid memory, APOE, and telomere data at baseline were included. Regression analyses were stratified by age group and relative status, adjusting for sex, education, and country. Among controls, ε2 was associated with better WM (p<0.05) in those aged 70-79. In relatives, ε2 was linked to better EM (p<0.05) in those 60-69. Within ε2 carriers, longer LTL related to higher EM/WM for those <60, but lower EM/WM among those 60-69 (p<0.05). In relatives, ε4 was linked to worse EM, but better WM in those <50. Within ε4 carriers ≥80, longer LTL related to poor EM/WM. Thus, APOE related differently to distinct memory functions, and such associations varied by familial longevity and age. LTL demonstrated both positive and negative associations with memory functions depending on APOE status and age group.

